# Genomic Organization and Developmental Expression of Glutathione *S*-Transferase Genes of the Diamondback Moth, *Plutella xylostella*

**DOI:** 10.1673/031.006.3501

**Published:** 2006-10-27

**Authors:** Shoji Sonoda, Muhammad Ashfaq, Hisaaki Tsumuki

**Affiliations:** Research Institute for Bioresources, Okayama University; Kurashiki, Okayama 710-0046, Japan

**Keywords:** copy number, gene expression, genomic sequence, molecular cloning, Lepidoptera

## Abstract

In the present study, we cloned and sequenced the entire coding regions of two glutathione *S*-transferase (GST) genes encoding PxGSTs and PxGSTe from the diamondback moth, *Plutella xylostella* L. (Lepidoptera: Yponomeutidae), along with their respective 5′ and 3′ flanking regions. The PxGSTs gene was composed of four exons and three introns. Based on amino acid identity with GST genes from other insects, this gene was classified as a member of the Sigma class. The gene encoding PxGSTe had an intron in the 5′ flanking region. Southern blot analysis showed that PxGSTs was a single copy gene, whereas there were homologous members of the PxGSTe gene in the *P*. *xylostella* genome. RNA gel blot analysis indicated that the expression levels of both genes changed with the developmental stage of *P*. *xylostella.*

## Introduction

The diamondback moth, *Plutella xylostella* L. (Lepidoptera: Yponomeutidae), is a serious pest of cruciferous vegetables worldwide (Talekar and Shelton 1993). *P. xylostella* has been shown to develop resistance to synthetic insecticides as well as to the biopesticide, *Bacillus thuringiensis* (Tabashnik 1994). Resistance to insecticides is generally conferred by metabolic detoxification of the insecticides, changes in nerve sensitivity or reduced cuticular penetration (Hemingway et al. 2004). In insecticide detoxification, involvement of three major groups of enzymes including carboxylesterases, cytochrome P450S and glutathione *S*-transferases (GSTs), have been reported (Hemingway et al. 2004).

GSTs are a family of proteins that have been demonstrated to be involved in detoxification of endogenous and xenobiotic compounds in vertebrates and invertebrates (Rushmore and Pickett 1993). GSTs have the capacity to conjugate reduced glutathione on the thiol of cysteine to various electrophiles and to bind with high affinity to a variety of hydrophobic compounds (Rushmore and Pickett 1993). In addition, some GSTs catalyze a dehydrochlorination reaction using reduced glutathione as a cofactor rather than a conjugate (Clark and Shamaan 1984). The cytosolic GSTs are dimeric enzymes approximately 25 kDa in size. Based on nucleotide similarities, Chelvanayagam et al. (2001) classified the insect GSTs into five classes: Delta, Omega, Sigma, Theta and Zeta. Lately, some GST genes from *Anopheles gambiae* have been assigned to Epsilon class (Ranson et al. 2002), although there are additional GSTs that cannot be assigned to any described class (Ding et al. 2003).

In *P*. *xylostella*, four GST isoenzymes and two GST genes have been reported so far (Chiang and Sun 1993; Ku et al. 1994; Huang et al. 1998; Eum et al., unpublished). In a previous study, we reported that a *P*. *xylostella* strain selected against chlorfluazuron showed higher GST activity and PxGSTe (formerly denoted as GST-3 by Huang et al. 1998) gene expression than the non-selected strain (Sonoda and Tsumuki 2005). In the present study, we isolated and determined complete nucleotide sequences of two GST genes encoding PxGSTs and PxGSTe and characterized their genomic organizations. Furthermore, we examined the developmental expression of these genes in *P*. *xylostella.*

## Materials and Methods

### Insects

Insects were obtained from a chlorfluazuron-resistant (CFR) *P*. *xylostella* colony maintained at 25° C under a long photoperiod (16L:8D) on radish seedlings. The CFR colony was established by selection of the non-selected strain with 5–10 ppm of chlorfluazuron in each generation (Sonoda and Tsumuki 2005).

### Amplification of genomic sequences by polymerase chain reaction (PCR)

**Figure 1.**
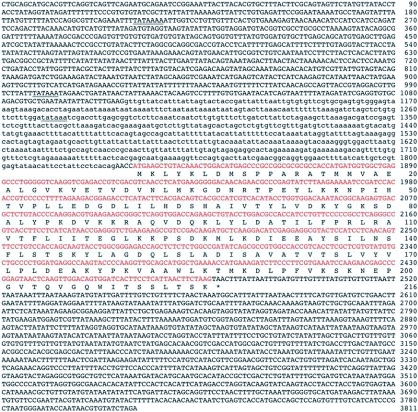
Nucleotide and deduced amino acid sequences of PxGSTe gene from *Plutella xylostella.* Putative TATA boxes are underlined. The asterisk indicates the translational termination codon. The coding sequences are shown by red. Nucleotides in lower case letters are intron sequences.

Genomic DNA extraction was described previously (Sonoda and Tsumuki 2005). The coding region of PxGSTe gene was amplified by PCR using a primer set, PxGSTe-5′-1, 5′-CTCACGAGCAATGAAAAGGTTCCAGTG-3′, (nucleotides 1,735–1,761 in Figure 1) and PxGSTe-3′-1, 5′-CAGCAGAATAATCCTTCCGCTTC-3′, (complementary to nucleotides 2,714–2,736 in Figure 1). Both primers were designed based on the published GST cDNA sequence (Huang et al. 1998) (GenBank/EMBL/DDBJ accession no. U66342). The coding region of PxGSTs gene was amplified by PCR using forward primer, PxGSTs-5′-1, 5′-GGCAT**ATGGCCAAGAAACTACACTACTTC**-3′, and reverse primer, PxGSTs-3′-1, 5′-CCGGATCC**TTATAGCGCGTAGACCTTCCTC**-3′. Both primers were designed based on the published GST cDNA sequence (Eum et al., unpublished) (accession # AB180447). On the 5′ ends, a restriction site for *Nde*I was added to PxGSTs-5′-1 and for *Bam*HI to PxGSTs-3′-1 as a requirement of another study. The coding sequences of PxGSTs-5′-1 (nucleotides 259–282 in Figure 3) and PxGSTs-3′-1 (complementary to nucleotides 2,574–2,595 in Figure 3) are shown as bold letters.

The 5′ and 3′ flanking regions of PxGSTe and PxGSTs genes were amplified by cassette-ligation based PCR amplification as described previously (Sonoda and Tsumuki 2005). All primers used for amplification of the 5′ and 3′ flanking regions of both GST genes were designed based on the genomic sequences mentioned above.

To clone the 5′ and 3′ flanking regions of PxGSTs gene, genomic DNA (1 µg) digested with *Hin*dIII was ligated with the *Hin*dIII cassette (Takara Bio, www.takara-bio.co.jp). PCR was performed using PxGSTs-3′-2, 5′-CCTCGTACAGGGGAAGCTGTCC-3′, (complementary to nucleotides 942–963 in Figure 3) and C1 (sequence included in the cassettes employed) for amplification of the 5′ flanking region. Subsequently, nested PCR was performed with PxGSTs-3′-3, 5′-GGCCATCCAGAGATCTGGTACC-3′, (complementary to nucleotides 353–374 in Figure 3) and C2 (sequence included in the cassettes employed). For amplification of the 3′ flanking region, we performed PCR with PxGSTs-5′-2, 5′-CCAGCTGACCTGGGCAGAATTC-3′, (nucleotides 2,424–2,445 in Figure 3) and C1. Nested PCR was performed with PxGSTs-5′-3, 5′-CTGTTCCTCGACTACGAGATCG-3′, (nucleotides 2,476–2,497) and C2.

*Pst*I digests ligated with the *Psf*I cassette (Takara Bio) were used for amplification of the 5′ flanking region of PxGSTe gene. PCR was performed using PxGSTe-3′-2, 5′-GGCCTCAGCCACCATCATGGTGGC-3′, (complementary to nucleotides 1,870–1,893 in Figure 1) and C1. Subsequently, nested PCR was performed with PxGSTe-3′-3, 5′-GCTCATGTCCAGTTTGTACAGC-3′, (complementary to nucleotides 1,836–1,857 in Figure 1) and C2. *Xba*I digests ligated with the *Xba*I cassette (Takara Bio) were used for amplification of the 3′ portion of PxGSTe gene. We performed PCR with PxGSTe-5′-2, 5′-CACTTCCTCATTAACTTCTAAGTAAC-3′, (nucleotides 2,457–2,482 in Figure 1) and C1. Nested PCR was performed with PxGSTe-5′-3, 5′-GATGTTTGTTTTATGTTGTTGTTAAT-3′, (nucleotides 2,494–2,519 in Figure 1) and C2.

PCR conditions were 30 cycles of 30 s at 94°C, 1 min at 65°C and 2 min at 72°C, followed by a final extension of 72°C for 7 min.

### Reverse transcription PCR (RT-PCR)

Total RNA extraction from whole body of fourth instar larvae and first strand cDNA synthesis were described previously (Tsukahara et al. 2003).

The coding region of PxGSTe cDNA was amplified by PCR using primers, PxGSTe-5′-1 and PxGSTe-3′-1. The 5′ and 3′ ends of PxGSTe cDNA were amplified by rapid amplification of cDNA ends (RACE) (Frohmann et al. 1988). For 3′ RACE, PCR was performed against the cDNA specified above using PxGSTe-5′-2 and M4 (Takara Bio). Subsequently, nested PCR was performed using PxGSTe-5′-3 and M4. cDNA was constructed from 1 µg of total RNA using a smart RACE cDNA amplification kit (Clontech, www.clontech.com) for 5′ RACE. PCR was performed against the cDNA using PxGSTe-3′-2 and the 10× universal primer (10× UPM) (Clontech). Subsequently, nested PCR was performed with PxGSTe-3′-3 and the nested universal primer (NUP) (Clontech).

**Figure 2.**
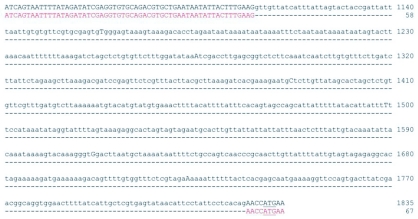
Alignment of the genomic sequence of the PxGSTe gene with the cDNA sequence. The initiation codon is underlined. The cDNA sequence is in red. Dashes indicate gaps in the cDNA sequence.

The coding region of PxGSTs cDNA was amplified by PCR using primers, PxGSTs-5′-1 and PxGSTs-3′-1. RACE was performed for amplification of 5′ and 3′ ends of PxGSTs cDNA. For 3′ RACE, 1st PCR performed with primer pair, PxGSTs-5′-2 and M4, was followed by 2nd PCR with PxGSTs-5′-3 and M4. For 5′ RACE, 1st PCR performed using PxGSTs-3′-2 and 10× UPM was followed by 2nd PCR using PxGSTs-3′-3 and NUP.

PCR conditions were 30 cycles of 30 s at 94°C, 1 min at 55°C and 1 min at 72°C, followed by a final extension of 72°C for 7 min.

### Cloning and nucleotide sequencing

The PCR-amplified fragments were cloned into pGEM-T Easy (Promega Corp., www.promega.com). Obtained clones were sequenced using a dye terminator cycle sequencing kit (Applied Biosystems, www.appliedbiosystems.com) with M13 forward and reverse primers by a DNA sequencer (Applied Biosystems, 3100 Avant Genetic Analyzer). Obtained sequences were analyzed using Genetyx-Mac ver. 10.1 (Software Development, www.sdc.co.jp).

### RNA gel blot analysis

Total RNA (20 µg) isolated from whole body extracts of different developmental stages were size-fractionated on a 1.2% agarose gel containing 0.66 M formaldehyde and transferred to a Biodyne PLUS membrane (Pall Corp., www.pall.com). The blot was hybridized with a random-primed 32P-labeled fragment containing the coding sequences of PxGSTe or PxGSTs gene (Figure 4) (Sambrook et al. 1989).

### Southern blot analysis

Genomic DNA (20 µg) digested with restriction enzymes were size-fractionated on a 1.5% agarose gel, transferred to a Biodyne PLUS membrane (Pall Corp.) and hybridized with a random-primed 32P-labeled probe, as specified above.

## Results

### Genomic sequence analysis of PxGSTe gene

Amplification of PxGSTe gene from genomic DNA by PCR using primers corresponding to the 5′ and 3′ ends of the cDNA sequence suggested that there are no introns in the coding region (data not shown). This was confirmed by nucleotide sequencing (Figure 1). To examine the genomic sequences that correspond to the 5′ and 3′ flanking regions of PxGSTe gene, cassette-ligation based PCR amplification was performed using genomic DNA. Amplified DNA fragments were cloned and sequenced. The combined genomic sequence of PxGSTe gene of 3,811 bp is shown in Figure 1 (accession # AB206478). Comparison of the genomic sequence to the cDNA sequence showed that there is an intron of 718 bp immediately preceding the start codon ATG (nucleotides 1,109–1,826 in Figures 1, 2 and 4). The ends of the putative intron were defined by GT-AG rule (Breathnach et al. 1978). Three putative TATA boxes were found at nucleotides 211–217 (TATAAAA), 998–1,003 (TATAAA) and 1,270–1,275 (TATAAA) (Figure 1). A search for the consensus sequences of CAAT (GGCCAATCT) and GC (GGGCGG) elements showed no exact match for the nucleotides within 1,830 bp of the 5′ flanking region. High AT content of 66.8% and 68.3% were found in the 5′ and 3′ flanking regions, respectively. The deduced amino acid sequence of the coding region showed 97.2% identity with that of the PxGSTe gene from the MPA strain of *P*. *xylostella* selected with methyl parathion (Huang et al. 1998). Differences were detected at amino acid positions 27, 38, 39, 116, 182 and 205. Among those, four at amino acid positions 27 (Asp to Glu), 38 (His to Asn), 116 (Phe to Ile) and 182 (Thr to Ala) were conservative (Figure 1 and data not shown).

**Figure 3.**
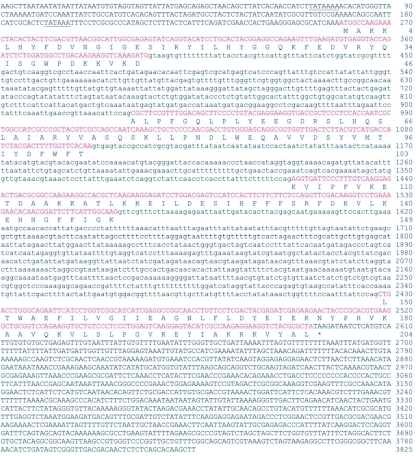
Nucleotide and deduced amino acid sequences of PxGSTs gene from *Plutella xylostella.* Putative TATA boxes are underlined. The asterisk indicates the translational termination codon. The coding sequences are shown in red. Nucleotides in lower case letters are intron sequences.

**Figure 4.**
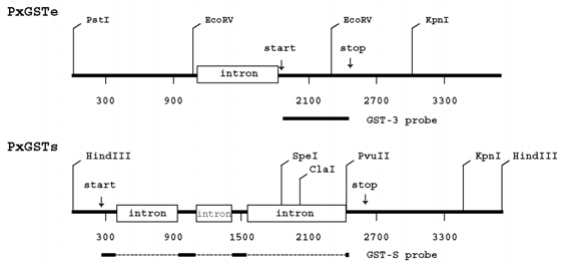
Restriction maps of PxGSTe and PxGSTs genes from *Plutella xylostella.* Probes for PxGSTe and PxGSTs genes are shown under restriction maps as solid bars.

### Genomic sequence analysis of PxGSTs gene

Amplification of the PxGSTs gene from genomic DNA using PCR primers corresponding to the 5′ and 3′ ends of the cDNA sequence produced a fragment, about 2.3 kb in length. The cDNA amplified by RT-PCR was about 600 bp in length, suggesting that there are introns in the coding region (data not shown). To examine the genomic organization of the PxGSTs gene, the nucleotide sequence of the amplified fragment from genomic DNA was compared with that of the cDNA. It was found that the PxGSTs gene is composed of four exons and three introns (Figures 3 and 4). Introns 1, 2 and 3 were identified at nucleotides 395–930 (536 bp), 1,102–1,417 (316 bp) and 1,558–2,427 (870 bp), respectively (Figures 3 and 4). The ends of the introns in PxGSTs gene were also defined by the GT-AG rule (Breathnach et al. 1978). The genomic sequences that correspond to the 5′ and 3′ flanking regions were amplified by cassette-ligation based PCR using genomic DNA. In the 5′ and 3′ flanking regions, no introns were predicted by comparison with the cDNA sequences (Figures 3 and 4). Combined genomic sequence of PxGSTs gene of 3,825 bp are shown in Figure 3 (accession # AB206477). The nucleotide sequences of the 5′ and 3′ flanking regions had AT content of 62.8% and 60.4%, respectively. Putative TATA boxes are identified at nucleotides 72–78 (TATAAAA) and 191–196 (TATAAA) (Figure 3). The other promoter elements such as CAAT element and GC element were not found within 258 bp of the 5′ flanking region. The genomic sequence contained an ORF of 612 bp. The deduced amino acid sequence was completely identical with that of the reported cDNA sequence (Eum et al., unpublished) (accession # AB180447).

### Southern blot analysis of GST genes

**Figure 5.**
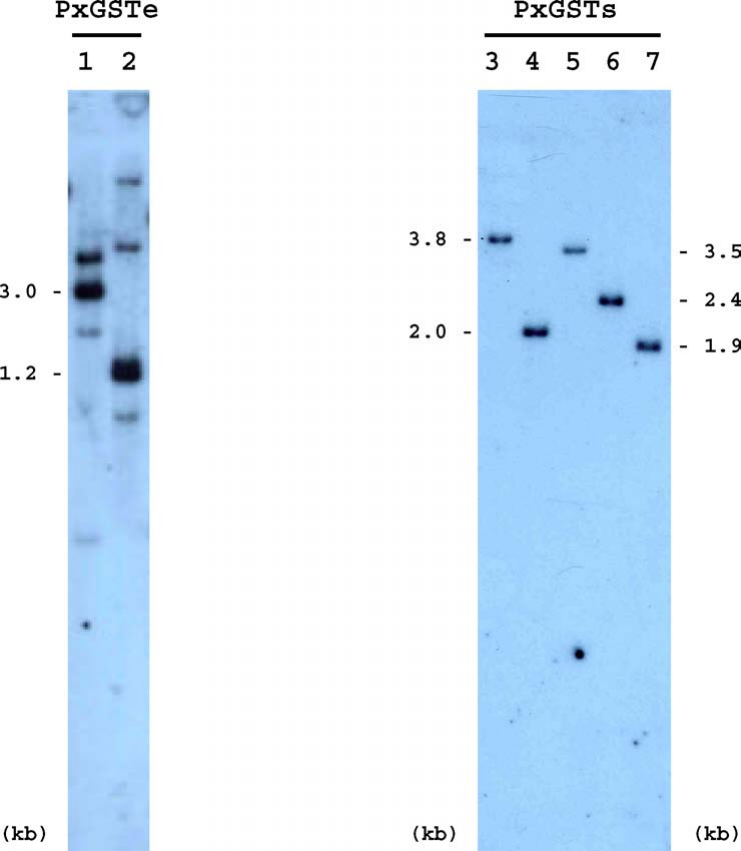
Southern blot analyses of PxGSTe and PxGSTs genes from *Plutella xylostella.* Total DNA digested with *Kpn*I (lane 1) or *Eco*RV (lane 2) in combination with *Pst*I was size-fractionated on an agarose gel, transferred to a nylon membrane and hybridized with a PxGSTe probe. *Hin*dIII (lane 3), *Hin*dIII/*Cla*I (lane 4), *Hin*dIII/*Kpn*I (lane 5), *Hin*dIII/*Pvu*II (lane 6) and *Hin*dIII/*Spe*I (lane 7) digests were similarly hybridized with a PxGSTs probe.

The presence or absence of genes homologous to PxGSTe or PxGSTs was examined by Southern blot analysis. Genomic DNA digested with *Kpn*I or *Eco*RV in combination with *Pst*I were hybridized with a PxGSTe gene fragment. In *Pst*I/*Kpn*I digests, a single intense band of 3.0 kb was observed as well as some less intense bands (Figure 5, lane 1). Similarly, a single intense band of 1.2 kb was observed in *Pst*I/*Eco*RV digests in addition to some less intense bands (Figure 5, lane 2). These results suggest that homologous members of the PxGSTe gene are present. When genomic DNA was digested with *Hin*dIII and probed with a PxGSTs fragment, a single intense band of 3.8 kb was observed (Figure 5, lane 3). Single intense bands of 2.0 kb, 3.5 kb, 2.4 kb and 1.9 kb were also detected in *Hin*dIII/*Cla*I*, Hin*dIII/*Kpn*I, *Hin*dIII/*Pvu*II and *Hin*dIII/*Spe*I digests, respectively (Figure 5, lanes 4 to 7). No additional strong or weak bands were detected in any of the digests (Figure 5 and data not shown), suggesting that PxGSTs is present in a single copy.

### Developmental expression of GST genes

**Figure 6.**
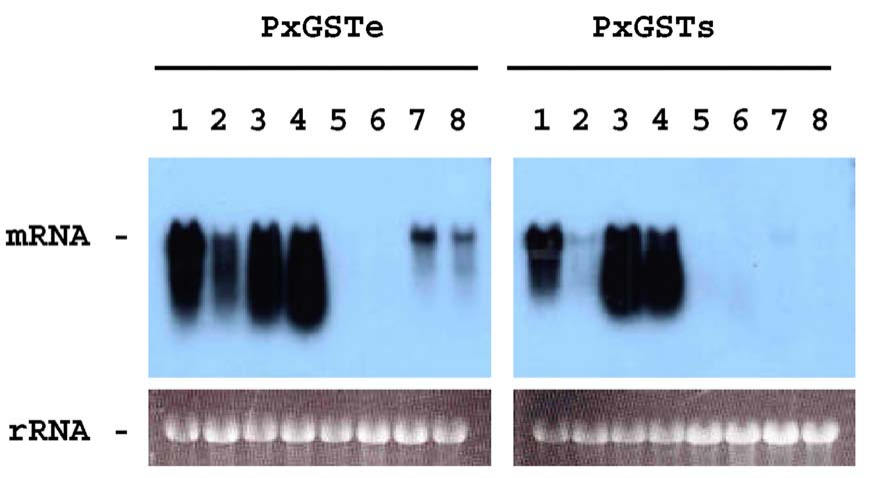
Developmental expression of PxGSTe and PxGSTs genes from *Plutella xylostella.* Total RNA extracted from 1st and 2nd instar larvae (lane 1), 3rd instar larvae (lane 2), male 4th instar larvae (lane 3), female 4th instar larvae (lane 4), male pupae (lane 5), female pupae (lane 6), male adults (lane 7) and female adults (lane 8) were analyzed by RNA gel blot analysis. The photographs of the ethidium bromide-stained RNA gel before transfer are also shown.

Developmental expression of PxGSTe gene was examined by RNA gel blot analysis (Figure 6). First and second instar larvae accumulated mRNA at very high levels. The mRNA levels apparently decreased in third instar larvae, and increased in the fourth instar, but little mRNA was detected in pupae. In adults, the PxGSTs mRNA levels increased again.

Expression of the PxGSTs gene in the course of development was also examined by RNA gel blot analysis (Figure 6). First and second instar larvae accumulated mRNA at high levels. In third instar larvae, low levels of mRNA were detected. The levels of mRNA increased in fourth instar larvae. Pupae and adults accumulated little PxGSTs mRNA.

In addition to the bands expected to be mRNA for PxGSTe and PxGSTs genes, smear signals were detected especially in fourth instar larvae (Figure 6). This might suggest the rapid turnover of the transcripts. However, we have no data supporting this hypothesis.

## Discussion

In mammalian glutathione *S*-transferases, sequence identities within the same gene class typically range from 60 to 80%, while inter-gene class identities are between 25 and 35%. The amino acid identity between the PxGSTe and PxGSTs genes was only 27.0%, suggesting that they belong to different gene classes. The amino acid sequence of the PxGSTs gene showed highest identity (68.6%) with the *Choristoneura fumiferana* GST (Feng et al. 1999). Since *C*. *fumiferana* GST was classified as a Sigma class (Enayati et al. 2005), PxGSTs from *P*. *xylostella* may be grouped in the same class. On the other hand, PxGSTe was classified as belonging to the Epsilon class (Enayati et al. 2005). However, the amino acid identity between PxGSTe and *Musca domestica* GST 6A (Wei et al. 2001), which belongs to the Epsilon class, was relatively low (47.6%). PxGSTe might belong to an as yet unrecognized GST class.

The coding region of PxGSTs gene was interrupted by three introns ranged from 316 bp to 870 bp. Intron 3 was a phase o intron that does not interrupt a codon, and the other two were phase 1 introns that exist between the first and second bases within a codon. In *A*. *gambiae*, two GST genes belonging to the Sigma class were reported (Ding et al. 2003). The coding region of one of these Sigma genes, *GSTs1-2*, was also interrupted by three introns. No other 27 GST genes in *A*. *gambiae* contain three introns, which supports the idea that the PxGSTs gene is a member of the Sigma class. The first intron, located approximately 50 amino acid residues from the N-terminal, is the splice site for the alternative transcripts of several *A. gambiae* GST genes including the Sigma class genes (Ding et al. 2003). At present, there is no information for alternative splicing of the PxGSTs gene. On the other hand, PxGSTe gene was intronless in the coding region, but the gene had an intron of 718 bp in the 5′ flanking region.

In *Drosophila melanogaster*, ten members of GST genes belonging to Epsilon class were clustered on chromosome 2R division 55C (Sawicki et al. 2003). Furthermore, in *A. gambiae*, a cluster of eight Epsilon GST genes was located in chromosome 3R division 33B (Ding et al. 2003). These observations in dipteran insects suggest that GST genes of the Epsilon class occurred via local duplication that led to independent expansions of the gene class. In the present study, the presence of homologous gene copies of the PxGSTe gene in the *P. xylostella* genome was demonstrated by Southern blot analysis. However, nucleotide sequencing of ca. 1.8 kb and ca. 1.3 kb corresponding to the 5′ and 3′ flanking regions of PxGSTe gene, respectively, revealed no homologous members in the regions. Extensive analyses of both flanking regions using genomic library of *P. xylostella* would be necessary to see if there are clusters of Epsilon GST genes in *P. xylostella.*

In general, actively feeding larvae are expected to have higher levels of GSTs, since they have the potential to take contaminating toxic chemicals along with the diet. For example, in *Aedes aegypti,* total GST activity increased in the course of larval development, peaked in pupae and then decreased in adult (Hazelton and Laing 1983). At the molecular level, it was revealed that 27 of 28 GST genes in *A. gambiae* are expressed in fourth instar larvae or one-day-old adults (Ding et al. 2003). On the other hand, in *C*. *fumiferana,* the GST gene belonging to the Sigma class are expressed at very low levels in feeding larvae but high levels in diapausing larvae (Feng et al. 1999). In the present study, expression levels of PxGSTe and PxGSTs genes fluctuated during the course of development. Furthermore, it was shown that expression patterns of both GST genes were independent of each other. For example, high levels of expression of the PxGSTe were observed in adults, while low levels of expression of the PxGSTs gene were found at this stage. These results suggest that both GST genes have different functions during development. It has been reported that GSTs are involved in protection of cells from oxidative stress (Daniel 1993) and the intracellular transport of hormones, endogenous metabolites and exogenous chemicals (Listowsky et al. 1988). Analysis of gene expression and protein activity using different tissues and organs is apparently necessary to elucidate the role of the GST genes in *P. xylostella.*

## Abbreviations

GST: glutathione S-transferase
RACE: rapid amplification of cDNA ends
